# Lipase improvement: goals and strategies

**DOI:** 10.5936/csbj.201209005

**Published:** 2012-10-15

**Authors:** Arnau Bassegoda, Silvia Cesarini, Pilar Diaz

**Affiliations:** a Department of Microbiology, University of Barcelona. Av. Diagonal 643, 08028-Barcelona. Spain

## Lipases for applied biocatalysis

Lipases have received great attention as industrial biocatalysts in areas like oils and fats processing, detergents, baking, cheese making, surface cleaning, or fine chemistry [1, 2]. They can catalyse reactions of insoluble substrates at the lipid-water interface, preserving their catalytic activity in organic solvents [3]. This makes of lipases powerful tools for catalysing not only hydrolysis, but also various reverse reactions such as esterification, transesterification, aminolysis, or thiotransesterifications in anhydrous organic solvents [4, 5]. Moreover, lipases catalyse reactions with high specificity, regio and enantioselectivity, becoming the most used enzymes in synthetic organic chemistry [6]. Therefore, they display important advantages over classical catalysts, as they can catalyse reactions with reduced side products, lowered waste treatment costs, and under mild temperature and pressure conditions [7]. Accordingly, the use of lipases holds a great promise for green and economical process chemistry [8, 9].

**Figure 1:**
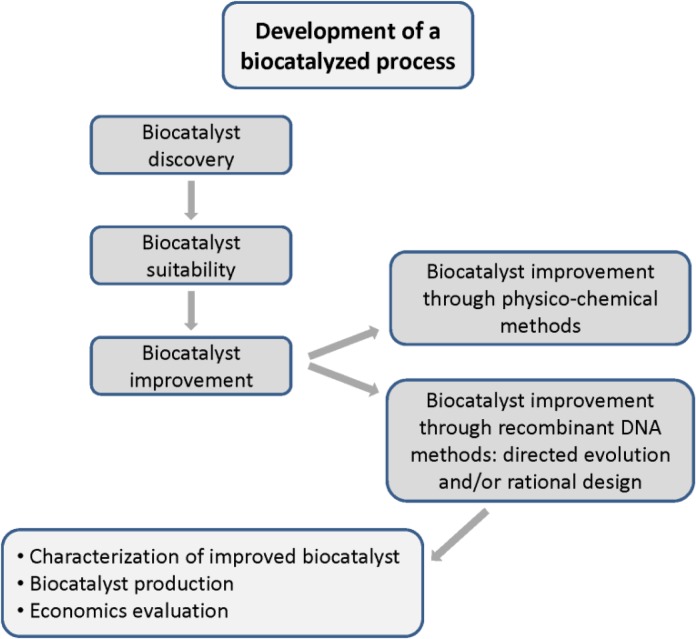
**Strategies that can be run to obtain better biocatalysts for commercial scale application**. Such strategies include the search for novel biocatalysts, and/or their improvement through immobilization or genetic modification to get the most suitable variant for each process using rational design or directed evolution.

However, performance of a lipase is not always sufficient for an industrial application [9] and most enzymes have sub-optimal properties for processing conditions [10]. In fact, there are still disproportionally few examples of commercial scale applications of such biocatalysts in the manufacture of fine chemicals. In order to improve enzyme-mediated process efficiency, two different pathways can be followed: *i*) fitting the process to the available biocatalyst by medium engineering or modification of the manufacturing system to suit the sensitivities of the biocatalyst [11], or *ii*) obtaining better biocatalysts through different strategies that can be run in parallel [9]. These strategies (Figure 1) include the exploration of biodiversity to expand the sources and number of new biocatalysts, immobilization of existing enzymes, reaction conditions modification [12, 13], or the proper modification of these biocatalysts to get the most suitable variant for a defined industrial process [9]. In this case the use of rational protein design to improve enzymes for which the 3D structure has been elucidated or homology-modelled [14], or the use of directed evolution can provide optimal biocatalysts [15].

## Lipase improvement

Rational protein design requires both, the availability of the structure of the enzyme and knowledge about the relationship between sequence, structure and mechanism-function. If the structural data of the enzyme are not available, the structure of a homologous enzyme can be used as a model [16, 17]. All this information can be used to identify specific residues that can be mutated in order to improve a specific property, such as substrate specificity or thermal robustness. The selected residues are then targeted for site-directed mutagenesis, and the variant expressed, purified and analyzed for the desired property [17, 18].

In contrast to rational protein design, directed evolution does not rely on a detailed understanding of the relationship between enzyme structure and function. It relies on the darwinian principles of mutation and selection [18]. In general terms, directed evolution consists on repeated cycles of random mutagenesis (and/or gene recombination) of a target gene, coupled with selection or high-throughput screening for isolation of the functionally improved variants. In this case, enough diversity can be created in the starting gene so that an improvement in the desired property will be represented in a library of variants. Subsequently, screening or selection methods are used to identify these variants, and then they are used as a template for the next generation of mutagenesis and selection [15, 19].

## Recent Strategies

**Figure 2:**
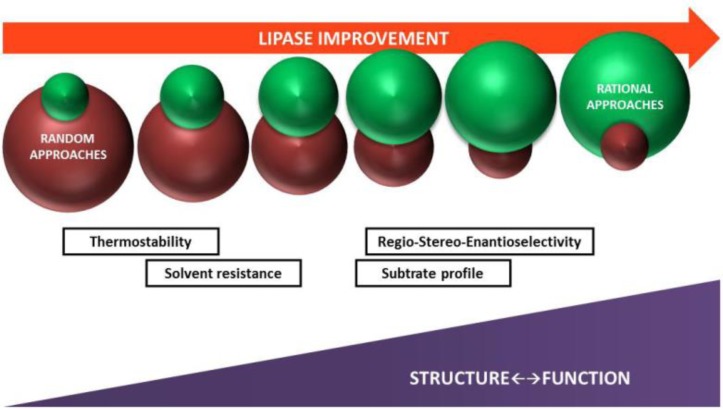
Correlation between structure-function knowledge and current protein engineering strategies to produce lipase variants with improved properties.

More recent developments have focused on making smaller libraries by using a combination of rational protein design and directed evolution procedures [20]. These methods use information based on enzyme structure, and target specific residues or regions on the protein in each evolution cycle (Figure 2). Based on this approach, saturation mutagenesis has been extensively used in recent enzyme improvement protocols. This method refers to the randomization of all amino acids at a defined position or to the simultaneous randomization of two or more positions in an enzyme [21, 22]. In this case the sequence space modified is smaller and therefore, faster to screen. Following this concept, Iterative Saturation Mutagenesis (ISM) was introduced as a new and more efficient method for directed evolution of functional enzymes [22, 23]. Based on a cartesian view of the protein structure, with iterative cycles of saturation mutagenesis applied at rationally chosen sites of an enzyme, this approach drastically reduces the necessary molecular biological work and the screening effort [24]. Both, rational protein design and directed evolution can be repeated or combined until the biocatalyst with the desired property is generated. Therefore, these protein engineering strategies have established as efficient tools to successfully improve biotechnologically relevant properties of enzymes [22, 24, 25]. More sophisticated approaches of such procedures have been developed by Verenium Co. that provide high throughput screening methods for Gene Site Saturation Mutagenesis (GSSM) [26] or for reassembly of the best properties of different genes through Tunable Gene Reassembly (TGR) technology [27]. Moreover, in recent years, computational protein design is getting more and more attention as a novel strategy to predict the effects of mutations on protein structure, function or stability of libraries of enzyme variants generated by means of in silico approaches [28].

The basis for choosing the sites for modification depends on the nature of the catalytic property to be improved [24, 29]. During the so called third wave of biocatalysts engineering, a drastic reduction of work and effort can be achieved [20], as more focused libraries reduce the amount of screening and can allow more complex assays that more closely reflect the final application conditions [11, 30]. In this context, for many chemical manufacturing processes the target functions for directed evolution are multidimensional: substrate specificity, activity, stabilities (thermal, pH, hydrophobicity, additives), and other parameters can be improved in a concerted effort to meet the process requirements [11, 31]. Furthermore, when a library fails to contain any hits, non-improved or even inferior mutants [32] could be used as templates in the continuation of the evolutionary pathway, thereby escaping from the local minimum [31].

To make a faster progress in protein engineering, a better understanding of how protein structure influences protein properties and a more critical evaluation of the many protein engineering approaches is needed [33, 34]. For this purpose, a set of tools have been designed for systematic analysis of the relationship between sequence, structure and function of lipases and related proteins: the Lipase Engineering Database (LED) [35], the Data Warehouse System (DWARF) [36] or the alpha/beta-Hydrolase Fold 3DM Database (ABHDB) [37]. These databases integrate sequence, structure and annotation information available in public databases like GenBank [38], PDB [39] and others, being useful tools to identify functionally relevant residues apart from the active site amino acids, and for the design of variants with improved properties [35]. With these improvements, the potential of lipases for chemical manufacturing can be fully acquired in the future.

## Improvement of thermal stability of lipases

Thermal stability is a major requirement for commercial enzymes, being critical for industrial applications, as thermal denaturation is a common cause of enzyme inactivation [40]. Attempting to meet the industrial demands, many lipases have been engineered to enhance their thermostability, including *Candida antarctica* lipase B [41, 42], *Rhizomucor miehei* lipase [43], or *Bacillus subtilis* lipase [44]. Several approaches can be applied to improve thermal stability: directed evolution with random mutagenesis based on error-prone PCR (*ep*-PCR), DNA shuffling, or the more recent iterative saturation mutagenesis guided by rational design. Factors generally known to enhance protein thermal stability involve the hydrophobicity profile, the number of hydrogen bonds, amino acid composition and their distribution or interactions in the protein [45]. Furthermore, a comparison between thermophile and mesophile homologues can help to perform predictions for stabilizing mutations [46]. Ahmad and collaborators demonstrated that minimal changes in the protein structure are sufficient for thermostability improvement, especially when changes take place on the protein surface [44]. However, correlation of thermostability with specific amino acids cannot be generalized [47]. Thus, although random mutagenesis requires a big screening effort, it seems to be the most popular approach for lipase thermostabilization (Figure 2). Ahmad and collaborators found a thermostable mutant of *B. subtilis* LipA after 7000 clones screened derived from *ep*-PCR. A variant with nine mutations was obtained showing a remarkable increase of 15°C in the melting temperature and a 20°C increase in optimum temperature compared to wild type lipase [44]. Error-prone PCR was also used by Sharma and collaborators to generate a highly thermostable mutant lipase starting from a metagenomic library: the improved mutant showed a 144-fold enhanced thermostability at 60°C [48]. Directed evolution by *ep*-PCR was also applied to stabilize the cold-active lipase from *Pseudomonas fragi* (PFL). After two rounds of evolution, a mutant with a 5-fold increase in half-life at 42°C and a shift of 10°C in optimum temperature was generated [49].

After introduction of iterative saturation mutagenesis for enzyme thermostabilization [22], the efficiency of thermal stability improvement has been enhanced and the screening effort dramatically facilitated (Figure 2). In addition, introduction of the B-factor criterion (B-FITTER) [29] to target the amino acid positions to modify [29] drastically increased the chances of success. A high average B-factor means that an amino acid residue has a low number of contacts with other amino acids, indicating a degree of flexibility that corresponds to low thermostability [50]. Using the two combined concepts, residues showing the highest B-factor of *B. subtilis* LipA were mutated producing two thermostable variant lipases with T_50_^60^ values of 89 and 93°C, respectively, in comparison to 48°C of the wild type enzyme [22]. More recently, the same approach was employed to obtain a *Rhizomucor miehei* lipase (RML) variant with good thermostability in a synthesis reaction: a double mutant was produced showing 63.1% residual activity after heating for 5h at 70°C, compared to 51.0% residual activity displayed by the wild type enzyme [51]. The same authors also reported the application of a high throughput screening method with a pH indicator based on synthetic activity instead of the more common lipolytic activity assay. In a recent work, structure comparison between homologous LipC (psychrophilic) and LipA (mesophilic) from *Pseudomonas* sp. 42A2, and application of the B-factor criterion allowed to identify the more flexible regions of LipC on a 3D model in order to increase thermostability of this lipase without affecting its psychrophilicity [52]. A mutant showing a 7-fold increased thermostability with respect to the wild type enzyme, but maintaining the cold-acting properties was obtained.

Despite the success of most enzyme thermostabilising assays performed in the last years, there is an increasing interest in understanding the causes that produce such stabilization. Circular dichroism spectroscopy can detect the unfolded structure of an enzyme as a function of temperature. Therefore, using this approach Sharma and collaborators reported that a thermostable mutant of *P. aeruginosa* lipase retained its secondary structure up to 70–80°C, whereas the wild type protein structure was completely distorted above 35°C [48]. Other authors have gone deeper in combining thermal inactivation profiles, circular dichroism, X-ray structure analyses, and NMR. These experiments revealed that mutation of surface residues hinder the tendency of *B. subtilis* LipA to undergo precipitation under thermal stress [53]. Although the mutant enzymes were obtained after application of the B-factor criterion, the results from physicochemical studies revealed that other factors seem to guide the outcome of the procedure as well, as for example, the screening method. As suggested, the cause for activity retention after heat shock in such B-factor-based thermostable mutants is the reduced precipitation of the unfolded intermediates rather than a really increased conformational stability [53].

Engineering disulfide bonds in protein folding is a promising strategy in rational protein design that has been applied to improve thermostability of different enzymes. Disulfide bonds make a significant contribution to the protein stability, mainly because of the decrease in the entropy of the unfolded form of proteins or the decrease in the unfolding rate of irreversibly denatured proteins [54]. Le and co-workers used two powerful computational tools (MODIP and DbD v1.20) to predict the possible disulfide bonds in CAL-B for enhancement of thermostability. Five residue pairs were chosen and mutated to cysteine. A CAL-B variant showed improved thermostability while maintaining its catalytic efficiency compared to that of the wild type enzyme. Additionally, the half-life at 50°C of the variant was 4.5-fold higher than that of CAL-B [55].

A successful example of computational protein design was reported by Ruslan and co-workers, who improved the thermostability of *Geobacillus zalihae* T1 lipase by introducing an ion-pair in the inter-loop [40]. Initially, the free energy change value and stability of putative mutants was predicted using computational algorithm tools, namely I-Mutant 2.0 [56]. The output data were then validated by a reliability index and finally, chosen residues were modified by site-directed mutagenesis.

## Solvent stability and structural features

Industrial application of lipases could be limited by their low stability and loss of activity in systems containing certain solvents [57]. Amino acids on the protein surface influence the solvent resistance of an enzyme and, modifying these residues, some goals have been achieved. For instance, by means of *ep*-PCR-directed evolution of *P. aeruginosa* LST-03 lipase, mutants with higher half life in DMSO, cyclohexane, *n*-octane, or *n*-decane were obtained [58]. The structural analysis of these variants revealed that 25% mutations were located on the surface of the enzyme. Substitution of superficial amino acid residues may be effective for preventing penetration of organic solvents into the protein molecule [59]. It was found that 50% of the released substitutions led to a shift to a higher *p*I value, which might be effective in keeping basic organic solvents, as DMSO, far from the lipase by ion repulsion. Moreover, addition of salt bridges, hydrogen bonds, and the additional packing of the hydrophobic core, all contributed to the observed increase of stability of these mutant lipases over the native protein in the presence of various organic solvents [60]. Formation of an extended communicating amino acid network on the surface of the enzyme, characterized by H-bonds and salt bridges, has also been reported by Reetz and collaborators as the main cause of stability increase of an enzyme [61]. In a recent work, the thermostable mutants of *B. subtilis* LipA obtained by iterative saturation mutagenesis [22] were characterized for their resistance to hostile organic solvents such as ACN, DMSO and DMF [62]. A correlation between the B-factors, originally used as thermostability criterion, and the robustness towards organic solvents was found. Assuming that in organic solvents most hydrophilic residues face towards the core and the hydrophobic ones are oriented towards the surface, an increase in surface hydrophobicity would appear that could facilitate lipase contact with organic substrates, thus enhancing activity in such media [63].

## Regio-, stereo- and enantioselectivity

Lipase stereoselectivity is due to the various diastereomeric interactions that occur between the stereoisomers and the active site of the enzyme. It depends largely on the substrate structure, interaction at the active site, and on the reaction conditions [64, 65]. Although all lipases show the same mechanisms for ester hydrolysis [66], the structures of their binding sites [67] and their stereopreferences are different, and their activity on defined substrates may vary. Taking advantage of the increasing structural data, molecular modelling strategies allow to study lipase-substrate interactions in each individual case at atomic level [68], proposing rational changes to promote the activity of one stereoisomer. The structure-based modification strategies have revealed that subtle changes in the protein can lead to great improvements in the regio- stereo- or enantioselectivity. A very interesting strategy was followed to modify *Candida albicans* lipase A (CAL-A) stereoselectivity and generate a variant with improved activity against *trans* fatty acid esters. Since the identification of residues involved in *cis*/*trans* double bond configuration is challenging, the amino acids forming the long acyl-binding tunnel were randomly mutated by site-directed saturation mutagenesis to generate twelve NDT-codon-based libraries. This strategy produced two CAL-A variants active only against *trans* fatty acid esters [69]. In another example, the molecular model of *Burkholderia cepacia* lipase docking the substrate (a bulky secondary alcohol) was used to detect two key residues interacting with the alcohol. Once detected, the residues were replaced by two sterically favourable amino acids, generating a mutant with excellent enantioselectivity for the kinetic resolution of bulky secondary alcohols [70].

On the other hand, randomness can also be introduced to selected residues. For instance, modelling a α-substituted *p*-nitrophenyl ester into the structure of CAL-A allowed identification of four residues whose side chains were pointing towards the substrate. Randomization of these residues led to generation of a CAL-A variant with optimum enantioselectivity for α-substituted *p*-nitrophenyl esters [71]. Two related works performed using *Yarrowia lipolytica* Lip2p for the hydrolysis of *2*-bromo-arylacetic acid esters showed that the enantioselectivity and enantiopreference of the enzyme is governed by two single residue positions. In a first work [72], a variant with improved *S*-selectivity was generated containing small amino acid residues at a defined position (232). This was enough to obtain a mutant with excellent enantioselectivity. On the other hand, bulky residues at that position and variants with single mutations in a second position (97) led to a mutant with slightly reversed enantiopreference. Later on, single mutations at the two positions producing reverse enantiopreference were combined rendering a totally *R*-selective variant with great activity and excellent enantioselectivity [73].

If introducing changes in the active site is an excellent strategy for enantioselectivity improvement, modification of the amino acids surrounding the active site can also lead to enzyme improvement. Schließmann and collaborators generated a mutant variant of *P. fluorescens* esterase with enhanced enantioselectivity in the kinetic resolution of bulky primary and secondary alcohols by applying combinatorial mutagenesis to the bulky residues forming a bottleneck at the entrance of the active site. Substitution of such bulky residues by less bulky amino acids resulted not only in a variant with increased enantioselectivity but also with higher conversion rates [74].

Structural knowledge of target enzymes has allowed development of more radical strategies resulting in rearrangements of the active site structure. Thus, a mutant variant of CAL-A with high enantioselectivity in the kinetic resolution of ibuprofen esters was generated by applying a structure-based combinatorial mutagenesis strategy. Docking the substrate into CAL-A model structure allowed detection of nine residues surrounding the substrate pocket. A simultaneous combinatorial mutagenesis employing binary amino acid sets was applied on these residues, obtaining a CAL-A mutant which combines 5 mutations that reshaped the substrate pocket thus displaying an increased activity and enantioselectivity [75]. A more drastic strategy was performed by Boersma and coworkers [76], who exchanged a loop near the active site entrance of *B. subtilis* LipA by loops from *F. solani* cutinase and *P. purpurogenum* acetylxylan esterase, obtaining LipA hybrids displaying inverted enantioselectivity in the kinetic resolution of 1,2-*O*-isopropylidene-*sn*-glycerol (IPG) esters. Moreover, the enantioselectivity of the most promising variant (cutinase hybrid) was further evolved by directed evolution [76]. Although the new mutant variants did not show synthetically useful enantioselectivity, the work provided novel perspectives on the evolution of lipases for increased properties.

When assays for enzyme improvement are coupled with extensive structure-function knowledge (Figure 2), new rational approaches for enantioselectivity optimization can arise. This was the case for several esterases aimed at the kinetic resolution of tertiary alcohols. Structure-function studies revealed that only esterases containing a GGG(A)X-type oxyanion hole can hydrolyse tertiary alcohol esters and how the amino acid composition of the oxyanion hole can greatly influence enantioselectivity [14, 35, 77, 78]. Thanks to the extensive structure-function data existing on this matter, it was possible to rationally transform esterase EstA from *Paenibacillus barcinonensis*, an enzyme showing very low conversion rates towards tertiary alcohols, into a synthetically useful biocatalyst with excellent enantioselectivity, without the need for experimental structural data [79]. A structure-based protein alignment guided the site-directed-mutagenesis of EstA oxyanion hole by introducing a motif previously associated with enantioselectivity [80–82], thus generating an EstA variant with excellent enantioselectivity [79].

## Substrate scope

As for enantioselectivity improvement, lipase substrate specificity can be drastically modified with few changes in the protein sequence, a very clear example of such strategy being the work performed with the metagenomic esterase Enzyme R.34 [83]. Despite applying random mutagenesis (*ep*-PCR), only one amino acid change converted the esterase into a triacylglycerol lipase. Based on a homology model, the authors hypothesized that the mutated amino acid forms a salt bridge with another residue that causes a distortion of the enzyme structure, thus exposing the catalytic site to larger substrates [83].

Knowledge of protein structures has provided crucial information for substrate specificity modification in order to adapt lipases to industrial process conditions. Based on *C. rugosa* LIP2 crystal structure, two residues located in the substrate binding pocket were identified and considered for saturation mutagenesis to investigate the impact of these amino acids on substrate specificity. Two mutant variants of the same position showed a shifted specificity from short- to medium/long-chain length triglycerides, revealing that such position has a big impact on substrate specificity. Replacement of the former small side chain group allowed accommodation of medium- to long-chain triglycerides in the substrate binding site [84]. A more accurate approach was used to rationally engineer *Candida rugosa* lipase (CRL) and CAL-A to obtain mutant variants with an altered substrate profile [85]. In both cases a careful study of the structural conformation of the scissile fatty acid binding site guided the rational strategy. CRL acyl-binding tunnel was blocked at different points by introducing bulky amino acids, thus generating mutant variants with different chain length specificity. In a similar way, the main acyl-binding tunnel of CAL-A was blocked in order to produce a small alternative pocket showing increased specificity for medium chain length fatty acids. The generation of CAL-A and CRL variants with altered substrate specificity confirmed the usefulness of the rational approaches [85, 86].

A remarkable work by Juhl and co-workers took full advantage of bioinformatics to generate a *C. antarctica* lipase B (CAL-B) variant accepting esters with branched and sterically demanding acids. An *in silico* library of 2400 CAL-B variants was built and screened *in silico* by substrate-imprinted docking. From the virtual screening, nine variants with single amino acid exchanges and increased activity were predicted and generated by site directed mutagenesis. Among the nine predicted variants only one displayed higher activity than the wild type against branched acids but not against sterically demanding acids. This work shows the potential of *in silico* approaches to predict mutant variants, thus reducing the time-consuming high throughput screening assays [87].

## Standing drawbacks

Despite the success of a large number of enzyme improvement studies, some designed modifications have failed to produce the lipase variant with the desired property. That was the case for *P. aeruginosa* LipA, a foldase-dependent enzyme for which obtaining an enzymatically active form in the absence of its chaperone would provide an easy and effective method for expression of such industrially interesting enzyme [88]. Although it was described that a single amino acid substitution (P112Q) in *Pseudomonas* sp. KFCC LipK allowed the lipase to spontaneously overcome the energy barrier of folding without participation of its cognate foldase [89], attempts to overcome this dependence in *P. aeruginosa* LipA failed to produce the desired variant. Substitution of amino acid 112 by ISM plus random mutagenesis was applied to LipA and the released mutants screened for activity in the absence of foldase. Unfortunately, no foldase-independent LipA variant was obtained [90], thus remaining yet a challenge for future strategy developments. To make a faster progress in protein engineering, a better understanding of how protein structure influences protein properties and a more critical evaluation of the many protein engineering approaches would be required [34].

## Summary and outlook

As shown above, there is a current task in protein engineering to design quality libraries containing a high proportion of active enzyme variants with improvements in the properties of interest [11].

The question of the optimal route of choice out of the many different possible pathways remains still unanswered and when a complete mutation scheme is considered, all pathways can be systematically explored for improvement of a desired property (Figure 2). However, when there is a good knowledge of the structure/function, the possibilities of success to get a variant with the desired properties increase notably and most pathways can provide better mutants with enhanced characteristics. These observations have ramifications for directed evolution in general and for evolutionary biological studies in which protein engineering techniques are applied. With these improvements, the potential of lipases for biotechnological manufacturing can be fully explored in the future.
